# Supplementary material for: Electrochemical Determination of Uric Acid Using a Nanocomposite Electrode with Molybdenum Disulfide/Multiwalled Carbon Nanotubes (MoS2@MWCNT)
Source: Nanomaterials (Basel). 2024 May 30;14(11):958. doi: 10.3390/nano14110958 (PMC11173421; doi:10.3390/nano14110958)
Supplement: Supplementary file 1 [file nanomaterials-14-00958-s001.zip › nanomaterials-2997527-supplementary.pdf]

Supporting Information

Electrochemical Determination of Uric Acid Using a Nanocomposite Electrode with Molybdenum Disulfide/Multiwalled Carbon Nanotubes (MoS_2@MWCNT)

Johisner Penagos-Llanos [1], Rodrigo Segura [1,*], Amaya Paz de la Vega [1], Bryan Pichun [1], Fabiana Liendo [1], Fernando Riesco [2], Edgar Nagles [2],*

1 Departamento de Química de los Materiales, Facultad de Química y Biología, Universidad de Santiago de Chile (USACH), Santiago 9170002, Chile

2 Facultad de Química e Ingeniería Química, Universidad Nacional Mayor de San Marcos, Lima 15081, Peru

* *Correspondence: enaglesv@unmsm.edu.pe¸ rodrigo.segura@usach.cl*

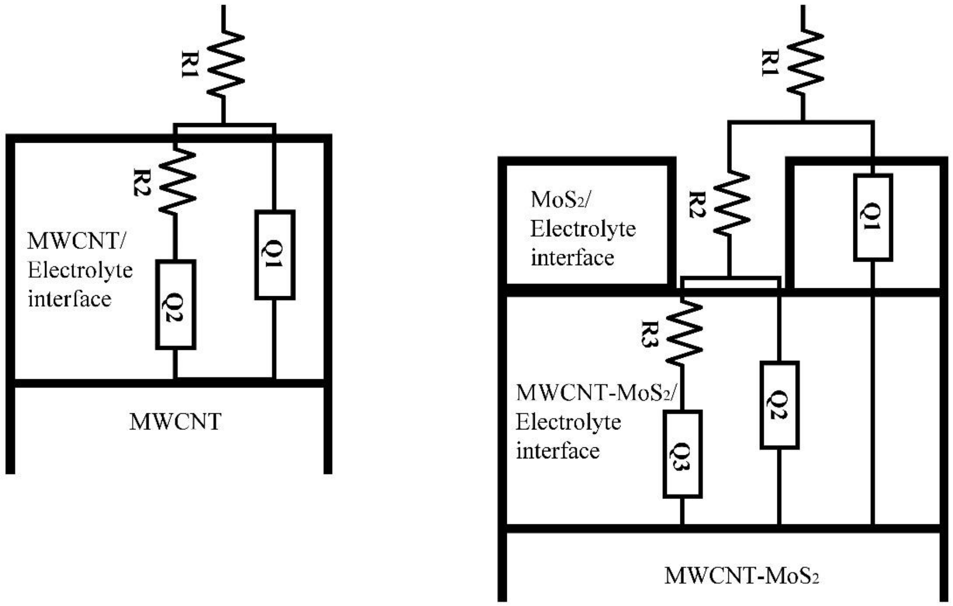

Figure S1. Physical model and equivalent circuits used for modelling the impedance spectra of MWCNT/E (left) and MoS_2@MWCNT/E (right)

Table S1. Circuit elements values of the modelled circuit obtained from the Nyquist diagram in Figure 2.

MWCNT/E				
Element	Parameter	Value	±	Unit
Q1	Q	1.48E-07	4.80E-08	s^{α}/Ω
Q1	α	0.901913	0.023951	
Q2	Q	0.000679	2.56E-05	s^{α}/Ω
Q2	α	0.277174	0.018258	
R1	R	0.01	0	Ω
R2	R	243.768	28.451	Ω
	X^2	0.085219		

MoS_2@MWCNT/E				
Element	Parameter	Value	±	Unit
Q1	Q	3.00E-06	1.28E-08	s^{α}/Ω
Q1	α	0.763707	0.000518	
Q2	Q	2.71E-05	2.02E-07	s^{α}/Ω
Q2	α	0.730907	0.00305	
Q3	Q	0.000565	1.76E-06	s^{α}/Ω
Q3	α	0.498799	0.001503	
R1	R	123.512	0.146919	Ω
R2	R	4060.52	11.7073	Ω
R3	R	2652.92	20.7554	Ω
	X^2	0.018148		
